# Comment on “Machine Learning for Early Detection of Hypoxic‑ischemic Brain Injury After Cardiac Arrest”

**DOI:** 10.1007/s12028-022-01526-y

**Published:** 2022-05-25

**Authors:** Noah S. Molinski, Aymen Meddeb, Martin Kenda, Michael Scheel

**Affiliations:** 1 Department of Neuroradiology, Charité-Universitätsmedizin Berlin, Freie Universität Berlin and Humboldt-Universität zu Berlin, Charitéplatz 1, 10117 Berlin, Germany; 2 Department of Radiology, Charité-Universitätsmedizin Berlin, Freie Universität Berlin and Humboldt-Universität zu Berlin, Hindenburgdamm 30, 12203 Berlin, Germany; 3 Department of Neurology, Charité-Universitätsmedizin Berlin, Freie Universität Berlin and Humboldt-Universität zu Berlin, Augustenburger Platz 1, 13353 Berlin, Germany

With great interest, we have read the article by Mansour et al. [1], reporting on the use of deep transfer learning to identify early signs of hypoxic-ischemic brain injury (HIBI) on head computed tomography (HCT) scans. The authors report a very high accuracy (0.94) of their model with respect to the detection of HIBI signs on HCT scans performed within hours after the return of spontaneous circulation. The authors conclude that “Deep transfer learning reliably identifies HIBI in normal appearing findings on HCT performed within 3 h after ROSC in comatose survivors of a cardiac arrest” [1]. This interpretation is likely too optimistic.

Deep learning networks show poor classification results and tend to be overfitted when trained on a very small data set [2]. A medical imaging data set of 54 HCT scans is a very small training data set. Further, we think that the following methodological issues could also contribute to overfitting in this study: (1) choice of the network, (2) the training pipeline (data augmentation, early stopping), and (3) principal component analysis (PCA) and repeated data usage.

No justification was given for why a VGG19 network was chosen, although it has a significantly worse accuracy in the analysis of CT data than, for instance, ResNet-50 or DenseNet-201 networks [3]. At the same time, it remains unclear why only ImageNet data and no medical imaging data were pretrained. The natural images from ImageNet differ in many aspects from clinical imaging data: image shape, colors, resolution, and dimension. Therefore, the network is trained on parameters that are irrelevant for its purpose, which may interfere with an accurate analysis.

Furthermore, it was not mentioned whether regularization methods such as transformations of the raw data (e.g., resizing, rotations, flipping, intensity shifting and/or scaling, Gaussian noise, zooming), weight constraints, or activity regularizations were used for reducing overfitting [4]. It remains unclear how many epochs the final model has been trained for. "Early stopping" (monitoring of the model performance on a validation set and then stopping training when the performance degrades) has become universally established to keep weights small during training and reduce the risk of overfitting [4].

Another aspect is the use of PCA. Because PCA is a linear algorithm for dimensionality reduction, the question arises on which basis a linear relationship between the detected features can be assumed. Given the complexity of the present data in terms of possible blurring or degradation due to fluctuating contrast, it is problematic to make such assumptions on the basis of the representation of shapes and images using smooth manifolds. Nonlinear methods (manifold learning), such as kernel PCA, t-distributed Stochastic Neighbor Embedding, or Multidimensional Scaling, could be applied instead. Moreover, the authors write “single-scan testing was repeated so that each of the 54 scans served as the test scan exactly one time” [1]. Although the leave-one-out cross validation described above improves model quality, the multiple repeated uses of the same data as training data can strongly facilitate overfitting.

As the authors reported that early HIBI signs were due to “subtle changes that evade the detection threshold of the human eye” [1], it would have been desirable to visualize by using heat maps or GradCAM, in which the subtle changes in the brain could start [5]. Those are important tools to plausibly illustrate the "thinking process of AI" to the readers.

The authors used a very small data set (*n* = 16) for validation. On this data set, the positive predictive value was 0.5, indicating that in the validation set a prediction of severe HIBI from early HCT had a 50% chance of being correct.

In conclusion, we agree that machine learning is an attractive new tool that may help to better predict severe HIBI from early HCT scans in cardiac arrest survivors in the future. The study by Mansour et al. [1] is a first step, but further studies on larger cohorts are necessary before it can be safely concluded that “deep transfer learning reliably identifies HIBI” from early HCT scans.
